# Executive Summary of the II Brazilian Guidelines for Atrial Fibrillation

**DOI:** 10.5935/abc.20160190

**Published:** 2016-12

**Authors:** Luiz Pereira de Magalhães, Marcio Jansen de Oliveira Figueiredo, Fatima Dumas Cintra, Eduardo Benchimol Saad, Ricardo Ryoshim Kuniyoshi, Adalberto Menezes Lorga Filho, Andre Luiz Buchele D'Avila, Angelo Amato Vincenzo de Paola, Carlos Antonio Abunader Kalil, Dalmo Antonio Ribeiro Moreira, Dario Celestino Sobral Filho, Eduardo Back Sternick, Francisco Carlos da Costa Darrieux, Guilherme Fenelon, Gustavo Glotz de Lima, Jacob Atié, José Carlos Pachón Mateos, José Marcos Moreira, José Tarcísio Medeiros de Vasconcelos, Leandro Ioschpe Zimerman, Luiz Roberto Leite da Silva, Marcio Augusto Silva, Mauricio Ibrahim Scanavacca, Olga Ferreira de Souza

**Affiliations:** 1 Sociedade Brasileira de Arritmias Cardíacas (SOBRAC), São Paulo, SP - Brazil

**Keywords:** Atrial Fibrillation/epidemiology, Anticoagulants, Catheter Ablation, Risk Factors, Sleep Apnea, Obstructive, Obesity, Genetic, Alcohol Drinking

## Introduction

Since 2009, when the Brazilian Society of Cardiology released the Brazilian Guidelines for Atrial Fibrillation,^1^ important studies on the subject have been published, particularly on new oral anticoagulants (NOACs). At least three of these drugs (dabigatran, rivaroxaban and apixaban) are currently approved for clinical use in Brazil.

In addition to pharmacological treatment, new data related to non-pharmacological treatment, notably the radiofrequency ablation (RA) procedure, have expanded the indication of this therapeutic approach. For this reason, an update of the guidelines is justified.

### Epidemiological changes in atrial fibrillation

In the last two decades, atrial fibrillation (AF) has become a public health problem, with high consumption of health resources. AF is the most frequent sustained arrhythmia in the clinical practice, with a prevalence of 0.5% - 1.0% in the general population. According to more recent studies, however, AF prevalence is almost two times higher than that in the last decade, ranging from 1.9% in Italy to 2.9% in Sweden, possibly associated with age increase.^2^ However, in addition to ageing, other potential factors may explain the increment in AF prevalence, including advances in the treatment of chronic heart diseases, leading to greater number of patients susceptible to AF. Furthermore, besides the classical risk factors for AF - hypertension, diabetes mellitus, heart valve disease, heart infarction and heart failure (HF)^3,4^ -new potential ones, including obstructive sleep apnea,^5^ obesity,^6^ alcohol consumption,^7^ physical exercise,^8^ family history and genetic factors,^9^ contribute to the increase in AF prevalence.

The most used AF classification in the clinical practice is based on its form of presentation. "Paroxysmal AF" is defined as an episode of AF that terminates spontaneously or with medical intervention within seven days of onset. The term "permanent AF" refers to AF episodes longer than seven days, and "long-term persistent AF" is used by some authors to refer to cases longer than one year. Finally, the term "permanent AF" is used when attempts to convert to sinus rhythm have been abandoned.

The prognosis of AF is related to its close association with increased risk of ischemic and hemorrhagic stroke, and mortality. Other important consequences of AF include cognitive changes and socioeconomic implications

### Prevention of thromboembolic phenomena

Patients with AF are more likely to have blood clots, which is an inherent risk of arrhythmia. Those at very low risk do not need anticoagulation, and should be identified and considered as non-eligible for this therapy. The score used for this purpose is the CHA2DS2-VASc (initials for *congestive HF, hypertension, age, diabetes mellitus, stroke, vascular disease, age, sex category*) (Table 1).^10^ Patients with a score of zero do not need anticoagulation, for the risk of thrombotic complications is very low. A CHA2DS2-VASc of 1 is considered a low risk (1.3% per year); in this case, anticoagulation is optional, depending on the risk of bleeding or patient's decision. All other patients have a definite indication for anticoagulation. HAS BLED (initials for hypertension, abnormal renal or liver function, stroke, bleeding, labile international normalized ratio - INR, elderly, drugs or alcohol use) is the most used score to estimate bleeding risk. (Table 2) A score > 3 indicates increased risk of bleeding by OACs. It is worth mentioning, however, that the score does not contraindicate the use of OACs, but rather gives direction on special measures aimed to make the treatment safer.

**Table 1:** (A) CHA2DS2-VASc score used to evaluate the risk of thromboembolic phenomena in patients with atrial fibrillation. (B) Adjusted annual event rate by score

[A]	
CHA_2_DS_2_-VASc	Score
**C**ongestive heart failure/left ventricular dysfunction	1
**H**ypertension	1
**A**ge ≥ 75 years	2
**D**iabetes mellitus	1
**S**troke/transient ischemic attack/thromboembolism	2
**V**ascular disease (prior myocardial infarction, peripheral artery disease or aortic plaque)	1
**A**ge 65–74 years	1
**S**ex **c**ategory (i.e. female gender)	1
	
[B]	
**Escore**	Adjusted stroke rate (%/year)*
0	0.0
1	1.3
2	2.2
3	3.2
4	4.0
5	6.7
6	9.8
7	9.6
8	6.7
9	15.2

**Table 2:** Clinical variables evaluated by the HAS-BLED score to identify patients at risk of bleeding induced by oral anticoagulants

HAS-BLED criteria	Score
Hypertension	1
Abnormal renal or liver function (1 point each)	1 or 2
Stroke	1
Bleeding	1
Labile (INR)	1
Elderly (e.g. age > 65 years)	1
Drugs or alcohol (1 point each)	1 or 2

INR: international normalized ratio.

There are four NOACs available for prevention of thromboembolic events: the direct factor Xa inhibitors rivaroxaban, apixaban and edoxaban and the direct fator IIa inhibitor dabigatran. Dabigatran was the first NOAC available at the market and validated by the RE-LY study (Randomized Evaluation of Long-term anticoagulant therapY with dabigatran etexilate.^11^ This is a prospective, randomized, phase III study that compared two doses of dabigatran (110 mg and 150 mg) twice a day with adjusted doses of warfarin. The primary outcomes were stroke and systemic embolism. Warfarin 150 mg showed better safety outcomes, including major bleeding, without statistical significance. The dose of 110mg was non-inferior to warfarin, showing a reduction of 20% in bleeding rate.

The ROCKET-AF (Rivaroxaban-once daily, oral, direct factor Xa inhibition compared with vitamin K antagonism for prevention of stroke and Embolism Trial in Atrial Fibrillation) study introduced rivaroxaban in clinical practice to prevent thromboembolic phenomena in patients with nonvalvular AF.^12^ This was a double-blind study, in which 14,264 patients at high risk for thromboembolic events were randomized to receive rivaroxaban or warfarin. The dose of rivaroxaban was 20 mg per day, or 15 mg in case of patients with kidney dysfunction received 15 mg. Rivaroxaban was non-inferior to warfarin on the primary outcomes (stroke and systemic embolism). With respect to safety outcomes, there was a significant decrease in the incidence of hemorrhagic stroke and intracranial hemorrhage, with no effect on mortality rate.

The ARISTOTLE (Apixaban for Reduction in Stroke and Other Thromboembolic Events in Atrial Fibrillation) was the main study on evaluation of apixaban in patients with nonvalvular AF.^13^ This randomized, double-blind study evaluated apixaban, given in 5mg doses twice a day or in adjusted dose of 2.5 mg, twice a day, in patients with at least two of the three following factors: age older than 80 years, body weight lower than 60 kg, and a serum creatinine level greater than or equal to 1.5 mg/dL . Warfarin was used as control. As compared with warfarin, apixaban significantly reduced the risk of the efficacy outcomes (stroke and systemic embolism) by 21%, major bleeding by 31%, and all-cause mortality by 11%.

Edoxaban was assessed in the ENGAGE -AF (Edoxaban versus Warfarin in Patients with Atrial Fibrillation) study.^14^ This was a three-arm, randomized, double-blind study on the use of warfarin and two regimens (low dose and high dose) of edoxaban. Both high-dose (60 mg once a day) and low-dose (30 mg once a day) edoxaban was non-inferior to warfarin. In patients assigned to receive edoxaban, the dose established at randomization was halved if any of the characteristics was present: creatinine clearance lower than 50 mL/minute, a body weight lower than 60 kg, or the concomitant use of a potent P-glycoprotein inhibitor (verapamil). High-dose edoxaban significantly reduced the rate of ischemic and hemorrhagic stroke, whereas a significant increase in ischemic stroke rate was observed in patients that received a low-dose of the drug. Therefore, the best efficacy-safety ratio was obtained from high-dose regimen. While the low-dose regimen of edoxaban provides higher safety in terms of the risk of major bleeding and hemorrhagic stroke, it tends to lose in efficacy.

Recommendations for prevention of thromboembolic phenomena in nonvalvular AF are described in Chart 1.

**Chart 1:** Recommendations for prevention of thromboembolic phenomena in nonvalvular atrial fibrillation

Recommendations	Class	Level of evidence
The CHA_2_DS_2_-VASc should be used in all patients	I	B
Patients at low risk, with a CHA_2_DS_2_-VASc of zero, have no indication of antithrombotic therapy	I	B
In patients with CHA_2_DS_2_-VASc of 1, the antithrombotic therapy may be indicated, taking into consideration the risk of bleeding and patients preferences	IIa	C
Patients with CHA_2_DS_2_-VASc ≥ 2 have an indication for antithrombotic therapy	I	A

The NOACs have caused a drastic change in the therapeutic approach to nonvalvular FA, in terms of prevention of thromboembolic events. However, drug-related hemorrhagic complications may represent a limitation. NOACs have short half-life, and hence a low-degree bleeding may be controlled by discontinuation of the drug. Different NOACs have distinct pharmacokinetic characteristics, which may influence the therapy. Dabigatran, for example, binds weakly to plasma proteins, and are potentially removed by hemodialysis. On the other hand, both riaroxaban and apixaban are not dialyzable, due to strong plasma protein binding. Activated charcoal could be used in case of anticoagulant ingestion within two hours of a hemorrhagic event, although its use is contraindicated in gastrointestinal bleeding. Activated charcoal is available in powder and may be diluted in water or juice for administration in awake patients or by nasogastric tube, at 1g/kg body weight. Despite not currently available in Brazil, there have been advances in medications that can reverse the effect of NOACs. Idarucizumab is a monoclonal antibody fragment that binds to dabigatran with higher affinity than to thrombin. The effect of idarucizumab as an anticoagulant reversal agent has been evaluated by intravenous administration; based on the results, the drug has been recently approved for clinical use in the United States.

 Andexanet is an inactive recombinant protein that reverses the anticoagulant effect by binding to activated factor X inhibitors (rivaroxaban, apixaban and edoxaban). The effect of its intravenous administration has been also evaluated, with satisfactory rates of reversal. It is expected that the use of andexanet in clinical practice will be approved soon.

Administration of supplemental clotting factors via frozen plasma may also be an option of anticoagulant reversal. However, the concentrations of these factors are lower than in prothrombin complex concentrates (PCC), which, in turn, may be indicated for severe hemorrhage.^15^

Although the OACs continue to be the main treatment option to prevent embolic phenomena in patients with AF, the use of anticoagulants is associated with risks, especially hemorrhagic stroke and other potentially severe bleeding, such as gastrointestinal bleeding. This therapeutic limitation, associated with the severity of AF-related embolic events, has motivated the development of new strategies aimed to reduce the incidence of thromboembolic phenomena. In this context, left atrial appendage closure (LAAC) emerged as an alternative approach. The main recommendations for this treatment strategy are described in Chart 2.

**Chart 2:** Recommendations for left atrial appendage closure

Recommendations	Class	Level of evidence
Patients at high risk for thromboembolic phenomena and with contraindication for oral anticoagulants	IIa	B
Patients with cardioembolic ischemic stroke despite correct use of oral anticoagulants	IIa	C

### Antiarrhythmic drugs in the clinical management of atrial fibrillation

When evaluating an AF patient, the patient may be allocated to a rhythm control or to a heart rate control strategy, depending on echocardiographic features and the progress in previous therapies. In this regard, the use of antiarrhythmic (AA) agents has a relevant role in both strategies. An initial assessment should identify the presence of structural heart disease, as well as to evaluate whether the cause is reversible.

There are a limited number of medications for the maintenance of sinus rhythm in Brazil. The available drugs are propaphenone, sotalol and amiodarone, and neither dofetilide nor droneadrone is available in the country. Propaphenone is useful for acute reversal and maintenance of sinus rhythm. It is a safe medication to be administered in patients with normal heart structure, but should be avoided in structural heart disease because of the risk of ventricular arrhythmia.^16^ Sotalol has shown no significant result in reversing arrhythmia acutely, but was effective in maintaining sinus rhythm in up to 72% of some groups of patients within 6 months, and thus may be useful in recurrence prevention. In addition, sotalol reduce the occurrence of symptoms by decreasing the ventricular response of the episodes due to its beta-blocker effect. The most common side effects are related to the beta-blocker effect, including tiredness and fatigue. Nevertheless, the most important symptom is prolongation of QT interval and development of torsade de pointes. Sotalol cannot be used in patients with congestive HF.^17^ Amiodarone is effective in reversing and maintaining sinus rhythm. Some studies have shown superiority of this drug over the others; however, in addition to the proarrhythmic risk, amiodarone may produce important side effects in many organs. Currently, it is the available drug for patients with congestive HF.^18^

Another strategy is the control of heart rate, which is important for both prevention of symptoms (e.g. palpitations, tiredness and reduced capacity for exercise), reduction of disease-related morbidity, and specially prevention of tachycardiomyopathy, which has an impact of patients' quality of life. However, the optimal heart rate in AF is still controversial. Many drugs have been tested and shown to be effective in the control of heart rate, including beta-blockers, non-dihydropyridine calcium channel blockers, and some antiarrhythmics, such as amiodarone and sotalol. To choose the most suitable drug, one must consider the severity of patients' symptoms, hemodynamic state, ventricular function, precipitating factors of AF and the risk for adverse events.

Beta-blockers are the most commonly used medications for the control of heart rate in AF.^19^ The main action is the blockade of adrenergic tone by competitive inhibition of the binding of cathecolamines to beta-receptors. This class of drugs mitigates the reduction in spontaneous depolarization (phase 4 of action potential), particularly in sinus node and atrioventricular (AV) node cells (reduces AV node conduction), and increases refractoriness of the His-Purkinje system. Non-dihydropyridine calcium channel blockers such as verapamil and diltiazem block L-type calcium channels especially in the AV node of cardiac conduction system. These drugs are effective in the control of heart rate in acute or permanent AF^20^ via intravenous or oral administration. Digoxin is commonly used in the control of heart rate in AF, although it is not considered a first line agent for this purpose. It has a direct action on the membrane of atrial cells, ventricular cells and conduction system, by increasing vagal tone, and consequently reducing sinus node automacity and AV node conduction. Recommendations for the use of antiarrhythmic drugs in AF are described in Chart 3.

**Chart 3:** Recommendations for catheter ablation of atrial fibrillation for maintenance of sinus rhythm

Recommendations	Class	Level of evidence
Symptomatic patients with paroxysmal AF refractory or intolerant to at least one class I/III AA drug when rhythm control is the strategy of choice	I	A
Symptomatic patients with AF refractory or intolerant to at least one class I or III antiarrhythmic drug	IIa	A
As first therapy in patients with symptomatic, recurrent AF (before AA drugs), if this is the patient’s preference	IIa	B
Symptomatic patients with long-standing persistent AF (>12 months), refractory or intolerant to at least one class I or III AA drug when rhythm control is the strategy of choice	IIb	B
As first therapy (before class I or III AA drug) in patients with persistent AF when rhythm control is the strategy of choice	IIb	C
Patients that cannot be treated with anticoagulants during or after the catheter ablation procedure	III	C

AF: atrial fibrillation; AA: antiarrhythmic

### Catheter ablation for atrial fibrillation

Intensive therapy by catheter ablation may be considered for rhythm control in AF.

### Heart rate control

In patients resistant or intolerant to medications for heart rate control, AV junction ablation (induction of complete AV block) with pacemaker implantation may be indicated.^21,22^ (Chart 4).

**Chart 4:** Recommendations for atrioventricular junction ablation in atrial fibrillation

Recommendations	Class	Level of evidence
AF affecting the therapy with ICD, in which other therapies could not be used or were not able to restore/maintain sinus rhythm or control the ventricular frequency	I	C
AF in patients with CRT for optimization of the therapy	IIa	B
AV node ablation with permanent ventricular stimulation is a reasonable strategy for heart rate control in cases when drug therapy is not suitable or when rhythm control is not possible	IIa	C
AV node ablation with permanent ventricular stimulation in clinically well patients	III	C

AF: atrial fibrillation; ICD: implantable cardioverter defibrillators; CRT: cardiac resynchronization therapy; AV: atrioventricular

This is a simple intervention with high success rate and low risk of complications, improving the quality of life of patients and reducing hospitalizations and HF incidence as compared with pharmacological treatments. Pacemaker implantation should be performed 4-6 weeks before the AV junction ablation for adequate maturation of electrode leads, since these patients are dependent on the pacemaker.

### Rhythm control

There is solid evidence that AF ablation (pulmonary vein isolation) is more effective than AA drugs in rhythm control,^23-25^ which has gradually increased the use of interventional therapy for AF. In recent international guidelines,^26-28^ ablation is recommended (Class I) in case of failure of an AA drug and also as the first choice (Class IIa) in patients with paroxysmal AF, without structural disease. Both patients with structural heart disease and patients with paroxysmal AF may be considered for ablation as the initial therapy, in case of suspicion of tachycardiomyopathy and patient's desire for this therapy.

Data confirming the benefits of AF ablation in very old patients, patients with long-standing persistent AF, or advanced HF are still missing.^28^ Its indication for asymptomatic patients has not been established yet, and is still a matter of controversy.^26-28^ There is still no evidence that AF ablation is a better intervention as compared with AA drugs with respect to reduction of hard outcomes, such as mortality, HF and stroke. These issues are being addressed by ongoing studies.^29^

The main objective of AF ablation is the electrical isolation of pulmonary veins. Among the available techniques, the most widely used is the conventional point-by-point radiofrequency (RF) ablation, guided by electroanatomical mapping and/or intracardiac electrocardiogram.^30-32^ The use of cryoablation balloon for circumferential ablation of pulmonary veins is an equally validated, alternative technique.^28,33,34^ Also, the use of circular multipolar catheters (that perform simultaneous delivery of energy through all electrodes^35^ and laser balloon catheters^36^ to create RF lesions has also increased.

Despite its proven efficacy, AF ablation is a high-complexity procedure that involves a nearly 4.5% risk for major complications.^28,37-39^ In addition, AF ablation is not a curative procedure. Recurrence is common, particularly following pulmonary vein reconnections or atrial substrate progression.^40^ In these cases, a new ablation procedure may be needed,^41^ and after ablation, all patients should be anticoagulated for a 2-3 month-period .^26-28^ At the end of this period, the anticoagulants may be suspended in patients with low risk of thromboembolic phenomena.^42,43^ Since late and asymptomatic recurrences of AF may also occur after ablation,^44,45^ patients should be monitored for a long period to ensure the control of arrhythmia.

Indications for AF are listed in Chart 3.

### New mapping and ablation technologies

Three-dimensional mapping of AF is nowadays considered the standard therapy for this condition worldwide. Aiming to visually guide the examiner in the analysis of left atrial anatomy and catheter localization, the technique allowed the reduction of radiation exposure for patients and staff.

### Three-dimensional mapping systems

The three-dimensional mapping systems allow a 3-D reconstruction of the left atrium and pulmonary veins by mobilization of a catheter positioned in heart chamber and in direct contact with the left atrial wall, with reduced X-ray exposition.^46^ There is a consensus among Brazilian experts that the use of tree-dimensional mapping increases the safety of ablation procedure.

### Intracardiac echocardiography

In intracardiac echocardiography, the catheter is placed inside the right atrium, corresponding to an optimal adjuvant strategy in ablation procedures.

### Rotational angiography

Rotational angiography is a x-ray method used for image acquisition of the left atrium in the electrophysiology laboratory using a basic hemodynamic system.^47,48^ The disadvantage of this method, as compared with the above described three-dimensional mapping technique, is the requirement of an ionic contrast media and a large amount of radiation.

### Ablation catheter technologies

Nowadays, nearly all procedures are performed using irrigated ablation catheters.^49,50^ More recently, irrigated ablation catheters with contact force sensor have become available, which measure the intensity of the interaction between the catheter and the myocardium, and may increase the efficacy of the lesion by reduction of complications.^51-54^

With respect to new energy sources, three types of sources are currently available - ultrasound, laser and cryotherapy.

### Robotic navigation technologies

Robotic navigation has emerged based on the high radiation exposure present in most AF catheter ablation modalities.^55-57^ However, studies demonstrating higher success or decreased complication rates with these technologies are not available yet, and their high cost is also a barrier to be overcome.

### Surgical treatment for atrial fibrillation

Many surgical procedures for the treatment of AF have been developed since the 80's. ^58-62^ The Cox-Maze III procedure, or labyrinth surgery, is the gold standard for surgical treatment of AF. The key components in this procedure and in most of the new surgical techniques for AF are also pulmonary vein isolation and atrial appendage resection.

Although the Maze surgery may be performed by a minimally invasive approach, involving a small chest incision, the technique requires 45-60 minutes of extracorporeal circulation (when performed by experienced hands) and cardioplegia.^63-65^ Furthermore, although this procedure may be performed alone, the surgery is commonly indicated for patients that require surgical interventions for other conditions, such as valvular and ischemic heart diseases.

Today, few patients are referred to surgery for AF alone. Even in those undergoing a surgical approach for other reasons, surgeons are reluctant to perform the Maze surgery, due to its complexity and magnitude.

### Hybrid treatment of atrial fibrillation

The so called "hybrid procedures" combine the minimally invasive epicardial surgery with electrophysiological mapping techniques and endocardial catheter ablation. This mixed approach is aimed to patients with persistent AF or long-standing persistent AF, to whom the use of one of these techniques alone would be unsatisfactory.^66-73^

In general, the initial results of hybrid procedures have been encouraging, especially considering the complexity of the treated population (persistent, long-standing AF). However, these results have been obtained from small samples. It is expected that the use of hybrid procedures expands as improvements in these techniques are made.

## References

[1] Zimerman LI, Fenelon G, Martinelli Filho M, Grupi C, Atié J, Lorga Filho A, Sociedade Brasileira de Cardiologia (2009). Diretrizes brasileiras de fibrilação
atrial. Arq Bras Cardiol.

[2] Zoni-Berisso M, Lercari F, Carazza T, Domenicucci S (2014). Epidemiology of atrial fibrillation: European
perspective. Clin Epidemiol.

[3] Benjamin EJ, Levy D, Vaziri SM, D'Agostino RB, Belanger AJ, Wolf PA (1994). Independent risk factors for atrial fibrillation in a
population-based cohort. The Framingham Heart Study. JAMA.

[4] Wang TJ, Larson MG, Levy D, Vasan RS, Leip EP, Wolf PA (2003). Temporal relations of atrial fibrillation and congestive heart
failure and their joint influence on mortality: the Framingham Heart
Study. Circulation.

[5] Gami AS, Hodge DO, Herges RM, Olson EJ, Nykodym J, Kara T (2007). Obstructive sleep apnea, obesity, and the risk of incident atrial
fibrillation. J Am Coll Cardiol.

[6] Frost L, Hune LJ, Vestergaard P (2005). Overweight and obesity as risk factors for atrial fibrillation or
flutter: the Danish Diet, Cancer, and Health Study. Am J Med.

[7] Conen D, Tedrow UB, Cook NR, Moorthy MV, Buring JE, Albert CM (2008). Alcohol consumption and risk of incident atrial fibrillation in
women. JAMA.

[8] Frost L, Frost P, Vestergaard P (2005). Work related physical activity and risk of a hospital discharge
diagnosis of atrial fibrillation or flutter: the Danish Diet, Cancer, and
Health Study. Occup Environ Med.

[9] Lubitz SA, Yin X, Fontes JD, Magnani JW, Rienstra M, Pai M (2010). Association between familial atrial fibrillation and risk of
new-onset atrial fibrillation. JAMA.

[10] Lip GY, Nieuwlaat R, Pisters R, Lane DA, Crijns HJ (2010). Refining clinical risk stratification for predicting stroke and
thromboembolism in atrial fibrillation using a novel risk factor-based
approach: the Euro Heart Survey on Atrial Fibrillation. Chest.

[11] Connolly SJ, Ezekowitz MD, Yusuf S, Eikelboom J, Oldgren J, Parekh A, RE-LY Steering Committee and Investigators (2009). Dabigatran vs. warfarin in patients with atrial
fibrillation. N Engl J Med.

[12] Patel MR, Mahaffey KW, Garg J, Pan G, Singer DE, Hacke W, ROCKET AF Investigators (2011). Rivaroxaban vs. warfarin in nonvalvular atrial
fibrillation. N Engl J Med.

[13] Granger CB, Alexander JH, McMurray JJ, Lopes RD, Hylek EM, Hanna M, ARISTOTLE Committees and Investigators (2011). Apixaban vs. warfarin in patients with atrial
fibrillation. N Engl J Med.

[14] Giugliano RP, Ruff CT, Braunwald E, Murphy SA, Wiviott SD, Halperin JL, ENGAGE AF-TIMI 48 Investigators (2013). Edoxaban versus warfarin in patients with atrial
fibrillation. N Engl J Med.

[15] Babilonia K, Trujillo T (2014). The role of prothrombin complex concentrates in reversal of
target specific anticoagulants. Thromb J.

[16] Freemantle N, Lafuente-Lafuente C, Mitchell S, Eckert L, Reynolds M (2011). Mixed treatment comparison of dronedarone, amiodarone, sotalol,
flecainide, and propafenone, for the management of atrial
fibrillation. Europace.

[17] Jull-Moller S, Edvardsson N, Rehenqvist-Ahlberg N (1990). Sotalol versus quinidine for the maintenance of sinus rhythm
after direct current conversion of atrial fibrillation. Circulation.

[18] Roy D, Talajic M, Dorian P, Connoly S, Eisenberg MJ, Green M (2000). Amiodarone to prevent recurrence of atrial
fibrillation. N Engl J Med.

[19] Olshansky B, Rosenfeld LE, Warner AL, Solomon AJ, O'Neill G, Sharma A (2004). The Atrial Fibrillation Follow-up Investigation of Rhythm
Management (AFFIRM) study: approaches to control rate in atrial
fibrillation. J Am Coll Cardiol.

[20] Ellenbogen KA, Dias VC, Plumb VJ, Heywood JT, Mirvis DM (1991). A placebo-controlled trial of continuous intravenous diltiazem
infusion for 24-hour heart rate control during atrial fibrillation and
atrial flutter: a multicenter study. J Am Coll Cardiol.

[21] Ozcan C, Jahangir A, Friedman PA, Patel PJ, Munger TM, Rea RF (2001). Long-term survival after ablation of the atrioventricular node
and implantation of a permanent pacemaker in patients with atrial
fibrillation. N Engl J Med.

[22] Wood MA, Brown-Mahoney C, Kay GN, Ellenbogen KA (2000). Clinical outcomes after ablation and pacing therapy for atrial
fibrillation: a meta-analysis. Circulation.

[23] Calkins H, Reynolds MR, Spector P, Sondhi M, Xu Y, Martin A (2009). Treatment of atrial fibrillation with antiarrhythmic drugs or
radiofrequency ablation: two systematic literature reviews and
meta-analyses. Circ Arrhythm Electrophysiol.

[24] Piccini JP, Lopes RD, Kong MH, Hasselblad V, Jackson K, Al-Khatib SM (2009). Pulmonary vein isolation for the maintenance of sinus rhythm in
patients with atrial fibrillation: a meta-analysis of randomized, controlled
trials. Circ Arrhythm Electrophysiol.

[25] Terasawa T, Balk EM, Chung M, Garlitski AC, Alsheikh-Ali AA, Lau J (2009). Systematic review: comparative effectiveness of radiofrequency
catheter ablation for atrial fibrillation. Ann Intern Med.

[26] Camm AJ, Kirchhof P, Lip GY, Schotten U, Savelieva E, Ernst S (2010). Guidelines for the management of atrial fibrillation: the Task
Force for the Management of Atrial Fibrillation of the European Society of
Cardiology (ESC). Eur Heart J.

[27] Calkins H, Kuck KH, Cappato R, Brugada J, Camm AJ, Chen SA (2012). 2012 HRS/EHRA/ECAS expert consensus statement on catheter and
surgical ablation of atrial fibrillation: recommendations for patient
selection, procedural techniques, patient management and follow-up,
definitions, endpoints, and research trial design: a report of the Heart
Rhythm Society (HRS) Task Force on Catheter and Surgical Ablation of Atrial
Fibrillation. Heart Rhythm.

[28] January CT, Wann LS, Alpert JS, Calkins H, Cigarroa JE, Cleveland Jr JC (2014). 2014 AHA/ACC/HRS Guideline for the Management of Patients With
Atrial Fibrillation: Executive Summary: a Report of the American College of
Cardiology/American Heart Association Task Force on Practice Guidelines and
the Heart Rhythm Society. Circulation.

[29] Packer DL, Lee KL, Mark DB, Kristi H (2010). Catheter Ablation vs Antiarrhythmic Drug Therapy for Atrial
Fibrillation: results of the CABANA.Pilot Study.

[30] Wilber DJ, Pappone C, Neuzil P, De Paola A, Marchlinski F, Natale A (2010). Comparison of antiarrhythmic drug therapy and radiofrequency
catheter ablation in patients with paroxysmal atrial fibrillation: a
randomized controlled trial. JAMA.

[31] Morillo CA, Verma A, Connolly SJ, Kuck KH, Nair GM, Champagne J (2014). Radiofrequency ablation vs antiarrhythmic drugs as first-line
treatment of paroxysmal atrial fibrillation (RAAFT-2): a randomized
trial. JAMA.

[32] Kim SS, Hijazi ZM, Lang RM, Knight BP (2009). The use of intracardiac echocardiography and other intracardiac
imaging tools to guide noncoronary cardiac interventions. J Am Coll Cardiol.

[33] Packer DL, Kowal RC, Wheelan KR, Irwin JM, Champagne J, Guerra PG (2013). Cryoballoon ablation of pulmonary veins for paroxysmal atrial
fibrillation: first results of the North American Arctic Front (STOP AF)
pivotal trial. J Am Coll Cardiol.

[34] Andrade JG, Khairy P, Guerra PG, Deyell MW, Richard L, Macie L (2011). Efficacy and safety of cryoballoon ablation for atrial
fibrillation: a systematic review of published studies. Heart Rhythm.

[35] Hummel J, Michaud G, Hoyt R, DeLurgio D, Rasekh A, Kusumoto F, TTOP-AF Investigators (2014). Phased RF ablation in persistent atrial
fibrillation. Heart Rhythm.

[36] Dukkipati SR, Kuck KH, Neuzil P, Woollett I, Kautzner J, McElderry HT (2013). Pulmonary vein isolation using a visually guided laser balloon
catheter: the first 200-patient multicenter clinical
experience. Circ Arrhythm Electrophysiol.

[37] Cappato R, Calkins H, Chen SA, Davies W, Iesaka Y, Kalman J (2010). Updated worldwide survey on the methods, efficacy, and safety of
catheter ablation for human atrial fibrillation. Circ Arrhythm Electrophysiol.

[38] Arbelo E, Brugada J, Hindricks G, Maggioni A, Tavazzi L, Vardas P (2012). ESC-EURObservational Research Programme: the Atrial Fibrillation
Ablation Pilot Study, conducted by the European Heart Rhythm
Association. Europace.

[39] Shah RU, Freeman JV, Shilane D, Wang PJ, Go AS, Hlatky MA (2012). Procedural complications, rehospitalizations, and repeat
procedures after catheter ablation for atrial fibrillation. J Am Coll Cardiol.

[40] Ouyang F, Tilz R, Chun J, Schmidt B, Wissner E, Zerm T (2010). Long-term results of catheter ablation in paroxysmal atrial
fibrillation: lessons from a 5-year follow-up. Circulation.

[41] Pokushalov E, Romanov A, De Melis M, Artyomenko S, Baranova V, Losik D (2013). Progression of atrial fibrillation after a failed initial
ablation procedure in patients with paroxysmal atrial fibrillation: a
randomized comparison of drug therapy versus reablation. Circ Arrhythm Electrophysiol.

[42] Themistoclakis S, Corrado A, Marchlinski FE, Jais P, Zado E, Rossillo A (2010). The risk of thromboembolism and need for oral anticoagulation
after successful atrial fibrillation ablation. J Am Coll Cardiol.

[43] Saad EB, D'Avila A, Costa IP, Aryana A, Slater C, Costa R (2011). Very low risk of thromboembolic events in patients undergoing
successful catheter ablation of atrial fibrillation with a CHADS2 score <
3: a long-term outcome study. Circ Arrhythm Electrophysiol.

[44] Wokhlu A, Hodge DO, Monahan KH, Asirvatham SJ, Friedman PA, Munger TM (2010). Long-term outcome of atrial fibrillation ablation: impact and
predictors of very late recurrence. J Cardiovasc Electrophysiol.

[45] Verma A, Champagne J, Sapp J, Essebag V, Novak P, Skanes A (2013). Discerning the incidence of symptomatic and asymptomatic episodes
of atrial fibrillation before and after catheter ablation (DISCERN AF): a
prospective, multicenter study. JAMA Intern Med.

[46] Rotter M, Takahashi Y, Sanders P, Haissaguerre M, Jais P, Hsu LF (2005). Reduction of fluoroscopy exposure and procedure duration during
ablation of atrial fibrillation using a novel anatomical navigation
system. Eur Heart J.

[47] Yeh DT, Oralkan O, Wygant IO, O'Donnell M, Khuri-Yakub BT (2006). 3D ultrasound imaging using a forward-looking CMUT ring array for
intravascular/intracardiac applications. IEEE Trans Ultrason Ferroelectr Freq Control.

[48] Singh SM, Heist EK, Donaldson DM, Collins RM, Chevalier J, Mela T (2008). Image integration using intracardiac ultrasound to guide catheter
ablation of atrial fibrillation. Heart Rhythm.

[49] Yamamoto T, Yamada T, Yoshida Y, Inden Y, Tsuboi N, Suzuki H (2014). Comparison of the change in the dimension of the pulmonary vein
ostia immediately after pulmonary vein isolation for atrial
fibrillation-open irrigated-tip catheters versus non-irrigated conventional
4 mm-tip catheters. J Interv Card Electrophysiol.

[50] Stabile G, Bertaglia E, Pappone A, Themistoclakis S, Tondo C, Calzolari V (2014). Low incidence of permanent complications during catheter ablation
for atrial fibrillation using open-irrigated catheters: a multicentre
registry. Europace.

[51] Okumura Y, Johnson SB, Bunch TJ, Henz BD, O'Brien CJ, Packer DL (2008). A systematical analysis of in vivo contact forces on virtual
catheter tip/tissue surface contact during cardiac mapping and
intervention. J Cardiovasc Electrophysiol.

[52] Yokoyama KN, Shah DC, Lambert H, Leo G, Aeby N, Ikeda A (2008). Novel contact force sensor incorporated in irrigated
radiofrequency ablation catheter predicts lesion size and incidence of steam
pop and thrombus. Circ Arrhythm Electrophysiol.

[53] Schmidt B, Kuck KH, Shah D, Reddy V, Saoudi N, Herrera C (2009). Toccata multi-center clinical study using irrigated ablation
catheter with integrated contact force sensor: first results. Heart Rhythm.

[54] Natale A, Reddy VY, Monir G, Wilber DJ, Lindsay BD, McElderry HT (2014). Paroxysmal AF catheter ablation with a contact force sensing
catheter: results of the prospective, multicenter SMART-AF
trial. J Am Coll Cardiol.

[55] Saliba W, Reddy VY, Wazni O, Cummings JE, Burkhardt JD, Haissaguerre M (2008). Atrial fibrillation ablation using a robotic catheter remote
control system: initial human experience and long-term follow-up
results. J Am Coll Cardiol.

[56] Pappone C, Vicedomini G, Manguso F, Gugliotta F, Mazzone P, Gulletta S (2006). Robotic magnetic navigation for atrial fibrillation
ablation. J Am Coll Cardiol.

[57] Di Biase L, Fahmy TS, Patel D, Bai R, Civello K, Wazni OM (2007). Remote magnetic navigation: human experience in pulmonary vein
ablation. J Am Coll Cardiol.

[58] Guden M, Akpinar B, Sanisoglu I, Sagbas E, Bayindir O (2002). Intraoperative salineirrigated radiofrequency modified Maze
procedure for atrial fibrillation. Ann Thorac Surg.

[59] Guiraudon GM, Campbell CS, Jones DL (1985). Combined sinoatrial node atrioventricular node isolation: a
surgical alternative to his bundle ablation in patients with atrial
fibrillation. Circulation.

[60] Cox JL, Schuessler RB, D'Agostino Jr HJ, Stone CM, Chang BC, Cain ME (1991). The surgical treatment of atrial fibrillation. III. Development
of a definitive surgical procedure. J Thorac Cardiovasc Surg.

[61] Cox JL, Boineau JP, Schuessler RB, Jaquiss RD, Lappas DG (1995). Modification of the Maze procedure for atrial flutter and atrial
fibrillation. I. Rationale and surgical results. J Thorac Cardiovasc Surg.

[62] Cox JL (2004). Cardiac surgery for arrhythmias. J Cardiovasc Electrophysiol.

[63] McCarthy PM, Gillinov AM, Castle L, Chung M, Cosgrove 3rd D (2000). The Cox-Maze procedure: the Cleveland Clinic
experience. Semin Thorac Cardiovasc Surg.

[64] Schaff HV, Dearani JA, Daly RC, Orszulak TA, Danielson GK (2002). Cox-Maze procedure for atrial fibrillation: Mayo Clinic
experience. Semin Thorac Cardiovasc Surg.

[65] Cox JL, Ad N, Palazzo T, Fitzpatrick S, Suyderhoud JP, Degroot KW (2000). Current status of the Maze procedure for the treatment of atrial
fibrillation. Semin Thorac Cardiovasc Surg.

[66] Robertson JO, Lawrance CP, Maniar HS, Damiano RJ (2013). Surgical techniques used for the treatment of atrial
fibrillation. Circ J.

[67] Gelsomino S, Van Breugel HN, Pison L, Parise O, Crijns HJ, Wellens F (2014). Hybrid thoracoscopic and transvenous catheter ablation of atrial
fibrillation. Eur J Cardiothorac Surg.

[68] Mahapatra S, LaPar DJ, Kamath S, Payne J, Bilchick KC, Mangrum JM (2011). Initial experience of sequential surgical epicardial catheter
endocardial ablation for persistent and long-standing persistent atrial
fibrillation with long-term follow-up. Ann Thorac Surg.

[69] Muneretto C, Bisleri G, Bontempi L, Curnis A (2012). Durable staged hybrid ablation with thoracoscopic and
percutaneous approach for treatment of long standing atrial fibrillation: a
30-month assessment with continuous monitoring. J Thorac Cardiovasc Surg.

[70] Bisleri G, Rosati F, Bontempi L, Curnis A, Muneretto C (2013). Hybrid approach for the treatment of long-standing persistent
atrial fibrillation: electrophysiological findings and clinical
results. Eur J Cardiothorac Surg.

[71] La Meir M (2014). Surgical options for treatment of atrial
fibrillation. Ann Cardiothorac Surg.

[72] La Meir M, Gelsomino S, Lucà F, Pison L, Parise O, Colella A (2013). Minimally invasive surgical treatment of lone atrial
fibrillation: early results of hybrid versus standard minimally invasive
approach employing radiofrequency sources. Int J Cardiol.

[73] Kurfirst V, Mokrácek A, Bulava A, Canádyová J, Hanis J, Pesl L (2014). Two-staged hybrid treatment of persistent atrial fibrillation:
short-term single centre results. Interact Cardiovasc Thorac Surg.

